# Enhancement of Li^+^ Transport Through Intermediate Phase in High-Content Inorganic Composite Quasi-Solid-State Electrolytes

**DOI:** 10.1007/s40820-025-01774-5

**Published:** 2025-06-11

**Authors:** Haoyang Yuan, Wenjun Lin, Changhao Tian, Mihaela Buga, Tao Huang, Aishui Yu

**Affiliations:** 1 Department of Chemistry, Collaborative Innovation Center of Chemistry for Energy Materials, Shanghai Key Laboratory of Molecular Catalysis and Innovative Materials, Institute of New Energy, Fudan University, Shanghai, 200438 People’s Republic of China; 2 Laboratory of Advanced Materials, Fudan University, Shanghai, 200438 People’s Republic of China; 3 ROM-EST Laboratory, ICSI Energy Department, National Research and Development Institute for Cryogenic and Isotopic Technologies − ICSI, 4 Uzinei, 240050 Ramnicu Valcea, Romania

**Keywords:** Quasi-solid-state electrolytes, Lithium metal anode, Intermediate phase, Na-superionic-conductors, 5 V-class cathode

## Abstract

Quasi-solid-state electrolytes, which integrate the safety characteristics of inorganic materials, the flexibility of polymers, and the high ionic conductivity of liquid electrolytes, represent a transitional solution for high-energy-density lithium batteries. However, the mechanisms by which inorganic fillers enhance multiphase interfacial conduction remain inadequately understood. In this work, we synthesized composite quasi-solid-state electrolytes with high inorganic content to investigate interfacial phenomena and achieve enhanced electrode interface stability. Li_1.3_Al_0.3_Ti_1.7_(PO_4_)_3_ particles, through surface anion anchoring, improve Li^+^ transference numbers and facilitate partial dissociation of solvated Li^+^ structures, resulting in superior ion transport kinetics that achieve an ionic conductivity of 0.51 mS cm^−1^ at room temperature. The high mass fraction of inorganic components additionally promotes the formation of more stable interfacial layers, enabling lithium-symmetric cells to operate without short-circuiting for 6000 h at 0.1 mA cm^−2^. Furthermore, this system demonstrates exceptional stability in 5 V-class lithium metal full cells, maintaining 80.5% capacity retention over 200 cycles at 0.5C. These findings guide the role of inorganic interfaces in composite electrolytes and demonstrate their potential for advancing high-voltage lithium battery technology.

**Supplementary Information:**

The online version contains supplementary material available at 10.1007/s40820-025-01774-5.

## Highlights

- A high-proportion inorganic composite quasi-solid-state electrolyte was fabricated through the integration of high-speed defoamed mixers with in situ polymerization methodology.
- The intermediate phase, which exhibits an affinity for anion adsorption, facilitates the partial dissociation of lithium-ion solvation structures, thereby enhancing transport kinetics.
- The exceptional interfacial stability was demonstrated through a lithium-symmetric cell operating without short-circuiting for 6000 h, while the 5 V-class lithium metal cell maintained 80.5% capacity retention after 200 cycles in 0.5C.

**Supplementary Information:**

The online version contains supplementary material available at 10.1007/s40820-025-01774-5.

## Graphical abstract

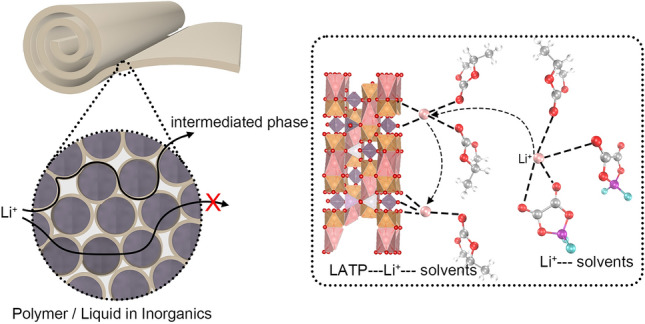

## Introduction

With an extraordinarily high specific capacity (3860 mAh g^−1^) and remarkably low reduction potential (− 3.04 V versus standard hydrogen electrode), lithium metal anodes contribute to the potential for achieving high energy density storage solutions that are crucial for next-generation clean energy storage systems [1–3]. However, the implementation of lithium metal anodes presents significant technical challenges, primarily stemming from their inherent high chemical reactivity. This reactivity poses substantial challenges for maintaining stable interfaces within the battery system. Traditional liquid electrolytes, despite their widespread use, carry inherent risks due to their toxicity and volatility [4]. In contrast, solid-state electrolytes serve as a safety enhancement through their ability to delay thermal runaway [5, 6].

When examining ionic conductivity at room temperature, these materials follow a hierarchical order: polymer electrolytes [7, 8], followed by oxides [9, 10], chlorides [11, 12], and sulfides [13, 14]. Polymer electrolytes, while benefiting from mechanical flexibility that aids in maintaining intimate electrode contact, are constrained by their relatively low ionic conductivity [8, 15]. Oxide electrolytes stand out for their superior thermodynamic stability, though their effectiveness is hampered by high grain boundary resistance that impedes ion transport [16]. Chlorides, despite their great conductivity, face compatibility challenges with lithium metal anodes, limiting their practical application [17, 18]. Sulfide electrolytes achieve the highest ionic conductivity among these categories but suffer from poor thermodynamic stability, making them vulnerable to decomposition [19, 20].

These inherent limitations inspired researchers to pursue composite approaches, combining different electrolyte types to create systems that capitalize on their complementary strengths while mitigating their weaknesses. One strategy involves incorporating inorganic particles as fillers within polymer matrices. This approach predominantly focused on materials like Na-superionic-conductor (NASICON) LATP (Li_1.3_Al_0.3_Ti_1.7_(PO_4_)_3_) [21, 22], LAGP (Li_1.5_Al_0.5_Ge_1.5_(PO_4_)_3_) [23], and garnet LLZTO (Li_6.5_La_3_Zr_1.5_Ta_0.5_O_12_) [24], chosen for their excellent thermodynamic stability and relatively straightforward synthesis procedures. Nevertheless, the ionic conductivity of oxide–polymer composite solid-state electrolytes remains predominantly limited by the polymer component’s inherent properties. The mechanism of Li^+^ transport within these polymer systems relies heavily on two factors: residual solvent content and segmental motion within regions of the polymer structure [25–28]. This understanding can lead to a solution that introduces small quantities of liquid electrolytes to create composite quasi-solid-state electrolytes. This strategy serves a dual purpose of enhancing interfacial contact by wetting electrode pore structures while simultaneously boosting bulk ionic conductivity [6, 29].

Polymer quasi-solid-state electrolytes through solution casting or in situ polymerization methods garnered extensive research attention [6, 30, 31]. A single step mixing process establishes liquid electrolyte as the primary conductive phase, and the polymer solid-state electrolyte enhances safety [31, 32]. The incorporation of inorganic materials into these systems presents an intriguing opportunity for further advancement. It could expand electrochemical stability windows, enhance mechanical robustness, and improve ionic conductivity [24, 33]. In providing comprehensive guidance for electrolyte design, the mechanism by which inorganic materials positively influence ionic conductivity remains incomplete. Recent investigations revealed that surface coupling interactions at the inorganic material interface play a vital role, yet several questions remain about how these interactions function.

These include understanding the impact of acid–base surface chemistry on the interfacial environment and determining how active versus inert oxides affect transport pathways. To amplify and investigate these interfacial coupling effects, our research utilized high proportions of inorganic fillers combined with a minimal amount of in situ polymerized polyethylene glycol diacrylate as a binding agent to fabricate composite quasi-solid-state electrolytes. This design strategy proved effective when implemented with LATP, achieving impressive performance metrics including room-temperature ionic conductivity of 0.51 mS cm^−1^ and an electrochemical window of 5.08 V. The enhanced performance can be attributed to several synergistic mechanisms. First, the acidic oxide interfaces selectively adsorb Lewis bases, which increases the Li^+^ transference number. Second, interfacial coupling at the inorganic material surfaces promotes partial dissociation of Li^+^ solvation structures, thereby accelerating ion transport kinetics. Third, the interfacial coordination environment provided by active inorganic materials appears particularly conducive to rapid ion conduction. These fundamental improvements in electrolyte properties translate into enhanced battery performance. In LiFePO_4_//Li full cells, the system maintains a substantial areal capacity of 2.42 mAh cm^−2^ even after 100 cycles. When implemented in 5 V-class LiNi_0.5_Mn_1.5_O_4_//Li full cells operating at 0.5C, the system retains 80.5% of its initial capacity after 200 cycles.

## Experimental Section

### Preparation of Quasi-Solid-State Electrolytes

A 2 mol L^−1^ LiDFOB (lithium difluoro(oxalate)borate, J&K scientific) liquid electrolyte was prepared by mixing high-boiling-point solvents PC (propylene carbonate, Adamas) and FEC (fluoroethylene carbonate, Adamas) in a 9:1 ratio. The in situ polymerization precursor solution was formulated by dissolving 0.5 wt% azobisisobutyronitrile (J&K scientific) in polyethylene glycol diacrylate (PEGDA, average Mn = 200, Aladdin). To fabricate PFE-TPDS, LATP (Li_1.3_Al_0.3_Ti_1.7_(PO_4_)_3_, 300 nm, Qingtao Energy Development Group Co., Ltd.), liquid electrolyte, and polymerization precursor solution were combined in a 6:3:1 mass ratio and homogeneously blended in a mixer to obtain a white slurry. The slurry was coated onto Al foil (16 μm) and covered with another Al foil layer to prevent electrolyte evaporation. *In situ* polymerization was conducted at 70 °C for 1 h to yield a flexible composite quasi-solid-state electrolyte. Alternative ratios (5:4:1, 7:2:1) were also investigated to determine optimal composition. For PFE-ALODS preparation, nano-alumina (30 nm, Macklin) was substituted for LATP as the inorganic component. Various surface-modified alumina materials were employed to produce: PFE-A-ALODS (acidic alumina, 200 mesh, Macklin), PFE-N-ALODS (neutral alumina, 200 mesh, Macklin), PFE-B-ALODS (basic alumina, 200 mesh, Macklin), and PFE-U-ALODS (Sub-nanometer alumina, 200–300 nm, J&K scientific).

### Electrochemical Characterization

Electrochemical impedance spectroscopy (EIS), linear sweep voltammetry (LSV), and lithium-ion transference number (${t}_{{\text{Li}}^{+}}$) measurements were taken using a Biologic VSP-300 electrochemical workstation. For ionic conductivity measurements, quasi-solid-state electrolytes of thickness (*d*) were assembled into symmetric cells using stainless-steel (SS) blocking electrodes. The ionic conductivity ($\sigma$) is calculated according to the following equation:

1 $$ {\upsigma } = d/RS $$

where *R* represents the electrochemical impedance of the SS symmetric cell and *S* denotes the surface area. LSV measurements were conducted using SS//Li half-cells at a scan rate of 0.1 mV s^−1^, with the electrochemical window determined at a current density threshold of 0.01 mA cm^−2^. Lithium-ion transference numbers were evaluated using Li//Li cells under DC polarization at ΔV = 10 mV, which is calculated using:

2 $$ t_{{{\text{Li}}^{ + } }} = I_{{\text{S}}} \left( {\Delta V - I_{0} R_{0} } \right)/I_{0} \left( {\Delta V - I_{{\text{S}}} R_{{\text{S}}} } \right) $$

where ${I}_{0}$ and ${I}_{\text{S}}$ represent the initial and steady-state currents, while ${R}_{0}$ and ${R}_{\text{S}}$ denote the initial and steady-state impedances.

Cycling tests were performed using a LAND-CT2001A system (Wuhan LANHE Technology Co., Ltd.). Cathode electrodes were prepared by homogeneously mixing active materials LFP (LiFePO_4_, 2.5–4.0 V *vs.* Li^+^/Li, 1 C = 170 mA g^−1^, Guangdong Canrd New Energy Technology Co., Ltd.), NCM622 (LiNi_0.6_Co_0.2_Mn_0.2_O_2_, 2.8–4.3 V *vs.* Li^+^/Li, 1 C = 180 mA g^−1^, Umicore Finland Oy), or LNMO (LiNi_0.5_Mn_1.5_O_4_, 3.5–5.0 V *vs.* Li^+^/Li, 1 C = 147 mA g^−1^, Ningbo Shanshan Co., Ltd.) with PVDF and Super P in an 8:1:1 mass ratio in NMP solvent. The resulting slurry was coated on Al foil with active material loading controlled at 2.5–3 mg cm^−2^. After drying at 80 °C for 12 h under vacuum, electrodes were cut into 12-mm-diameter disks. CR2025-type coin cells were assembled by stacking cathode, quasi-solid-state electrolyte (16 mm diameter), and lithium metal anode (15 mm diameter and 1 mm thickness), followed by sealing in an Ar-filled glove box. In situ impedance measurements were conducted on LNMO//Li cells at 0.2C with impedance measured every 30 min during charge–discharge cycles. For impedance evolution studies, measurements were taken after each discharge, with distribution of relaxation times (DRT) analysis performed using DRTtools transformation of galvanostatic electrochemical impedance spectroscopy (GEIS) data [34].

## Results and Discussion

### Surface Acid–Base Characteristics

**Fig. 1:**
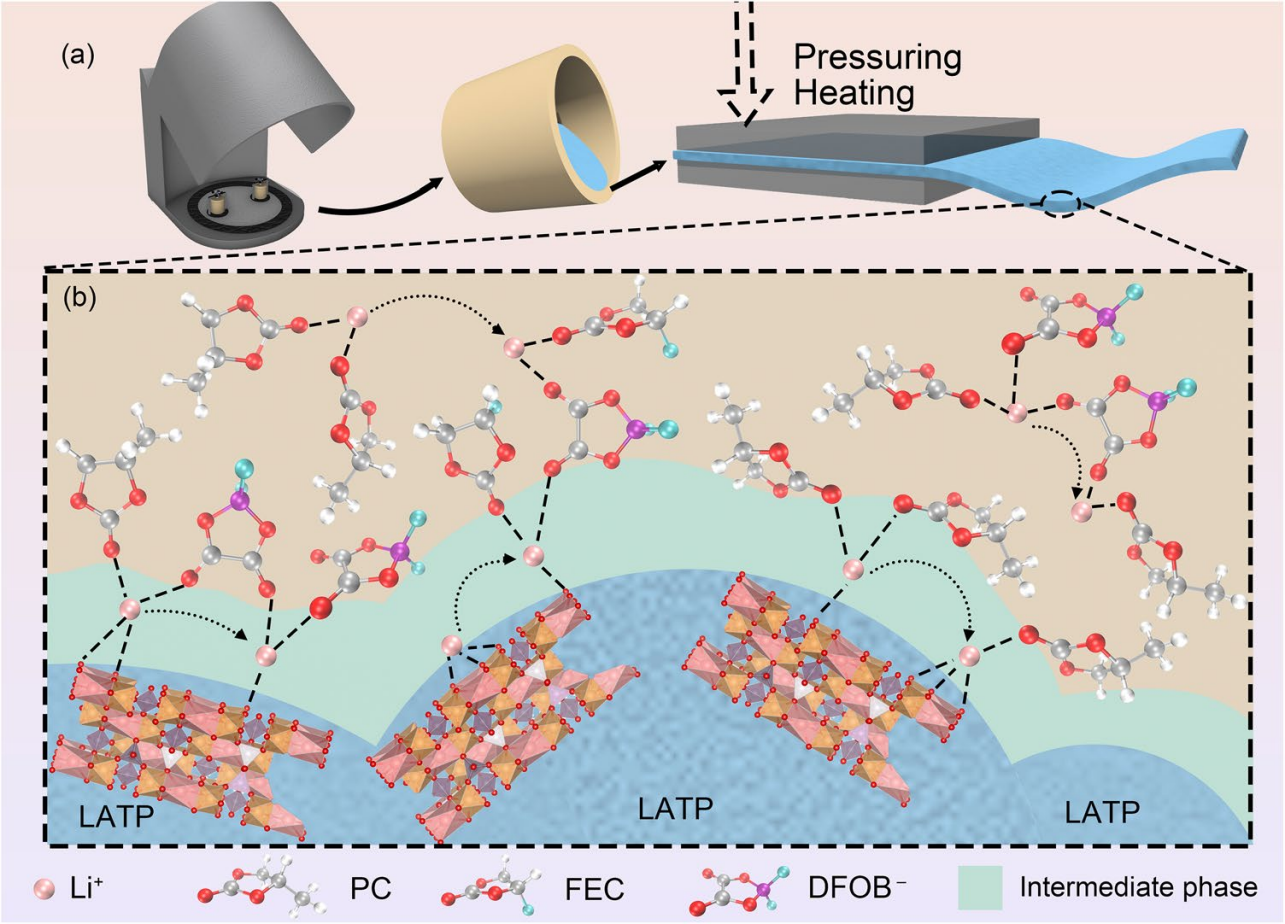
**a** Schematic diagram of the preparation process of the flexible membrane. **b** Schematic diagram of the conduction mechanism of composite solid electrolyte

As illustrated in Fig. 1a, inspired by the in situ polymerization methodology, the precursor slurry, which had been thoroughly homogenized through ball milling, was subsequently utilized to fabricate flexible composite quasi-solid-state electrolyte membranes (Figs. S1b and S2). The optimization of proportions between inorganic material, high-temperature-resistant liquid electrolyte (PFE), and in situ polymerized polymer (DS) was accomplished through synthetic methodology control, which aimed to determine both the maximum achievable inorganic and the minimum required electrolyte that would still maintain film-forming capabilities (Fig. S1a–c). Within such a complex multiphase system, multiple ionic conduction mechanisms inevitably coexist and operate simultaneously. These diverse transportation pathways encompass coordinated conduction through the liquid electrolyte, vacancy hopping within the inorganic solid-state electrolyte, interfacial interactions between different phases, and limited polymer chain segment mobility (Fig. 1b).

**Fig. 2:**
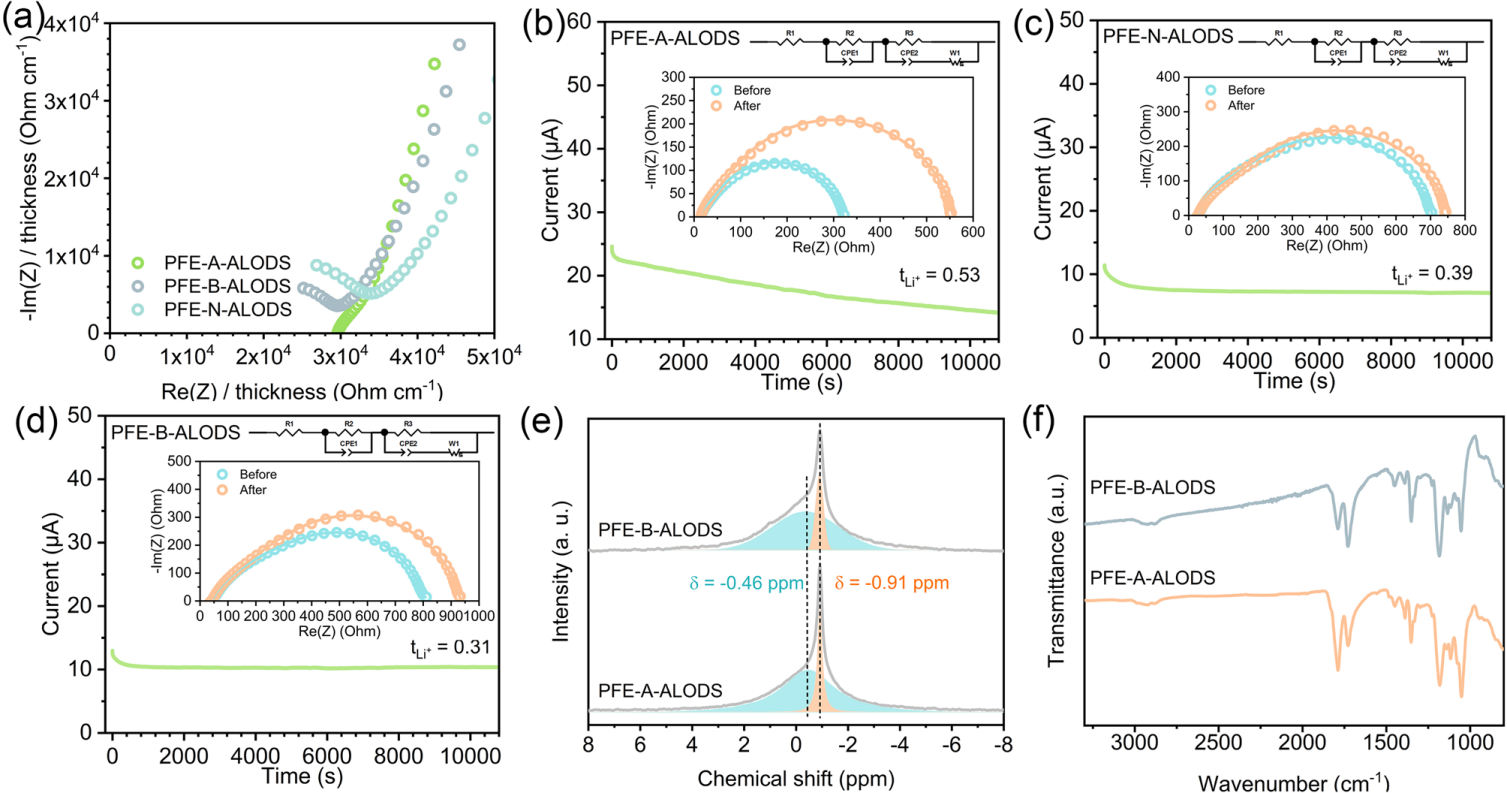
**a** Electrochemical impedance spectra of stainless-steel symmetric cells at the unit thickness in 25 °C. Current changes during constant voltage polarization in a lithium-symmetric battery with **b** PFE-A-ALODS, **c** PFE-N-ALODS, and **d** PFE-B-ALODS (The inserted figure is the impedance before and after the test, where the line is the equivalent circuit-fitting result). **e** ^7^Li solid-state NMR of PFE-A-ALODS and PFE-B-ALODS. **f** FT-IR spectra of PFE-A-ALODS and PFE-B-ALODS after cycling

In this system, where inorganic oxide material (Fig. S3) substantially predominates over organic polymer, the interaction between the two highly conductive phases, including the inorganic material and the liquid electrolyte, perhaps emerges as the primary factor governing Li^+^ conduction. The acid–base properties of their surfaces emerge as another crucial factor influencing ionic conductivity. While maintaining comparable ionic conductivity levels, the heterogeneous surface modification of Na-superionic-conductor (NASICON) LATP (Li_1.3_Al_0.3_Ti_1.7_(PO_4_)_3_) poses challenges. Valuable insights can be derived from examining alumina samples with varying surface acid–base characteristics. Although the acidic, neutral, and basic alumina (named PFE-A-ALODS, PFE-N-ALODS, and PFE-B-ALODS, respectively) exhibit smaller specific surface areas than nano-alumina, there are lower ionic conductivities where the overall ionic conductivity differences among these variants remain minimal (0.015–0.016 mS cm^−1^, Fig. 2a). This phenomenon can be elucidated through the Lewis acid–base pair theory that basic surfaces preferentially adsorb Lewis acidic cations (predominantly Li^+^). In contrast, acidic interfaces show greater affinity for lithium salt anions possessing higher electron density [35, 36]. Consequently, acidic interfaces may facilitate enhanced lithium salt dissociation, thereby indirectly promoting rapid lithium-ion transport. The experimental results depicted in Fig. 2b–d provide compelling evidence supporting our hypothesis regarding selective adsorption at interfaces with different acid–base properties. The Li^+^ transference number (${\text{t}}_{{\text{Li}}^{+}}$) demonstrates a remarkable progressive increase from 0.31 to 0.39, and ultimately to 0.53 as surface acidity intensifies, which correlates with surface-specific anchoring.

Nuclear magnetic resonance (NMR) spectroscopy, particularly under magic angle spinning (MAS) conditions applied to the solid state, provides direct insights into the chemical environments of Li^+^ through ^7^Li signal detection. The analysis reveals several distinct chemical environments for Li^+^ within these complex systems. In the pristine electrolyte, Li^+^ exists in solvated structures characterized by a chemical shift at – 0.47 ppm (Fig. S4). When the electrolyte interacts with the polymer matrix, Li^+^ encapsulated within the polymer structure exhibits a chemical shift of approximately − 0.60 ppm, indicating a modified chemical environment due to polymer–liquid interactions. The reduced surface area results in a lower proportion of interface-adsorbed species, again suggesting that the interface-mediated coordination structures play a more crucial role in Li^+^ transport than previously anticipated. The variation in surface acid–base properties does not fundamentally alter the Li^+^ coordination modes (Fig. 2e). The − 0.45 ppm signal can be attributed to Li^+^ that reverted to their solvated state within the electrolyte, having dissociated from the polymer matrix. Importantly, this rules out the possibility of new electrolyte–polymer combination structures forming during cycling, so the emergence of a signal at − 0.91 ppm most likely indicates Li^+^ interacting with inorganic interfaces.

The Fourier transform infrared spectroscopy (FT-IR) analysis provides comprehensive insights into the solvation structures and their evolution during electrochemical cycling. PFE-A-ALODS and PFE-B-ALODS have similar interaction patterns, but there is a larger peak area at 1650 cm^−1^ in PFE-B-ALODS compared to PFE-A-ALODS in Fig. 2f. This difference arises because PFE-A-ALODS, with its greater affinity for anions, draws fewer Li^+^ (and consequently fewer solvent molecules) to its surface. More solvent molecules near the basic surface of PFE-B-ALODS result in more carbonyl groups experiencing the interface-induced environment, producing a stronger spectral signature at 1650 cm^−1^.

### Specific Surface Area

**Fig. 3:**
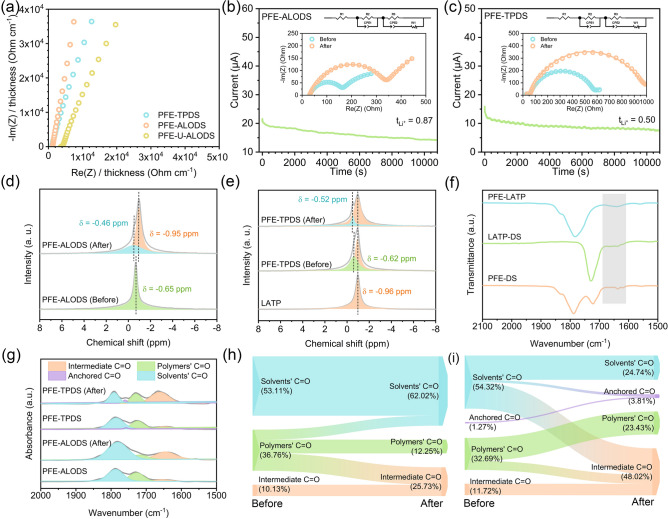
**a** Electrochemical impedance spectra of stainless-steel symmetric cells at the unit thickness in 25 ℃. Current changes during constant voltage polarization in a lithium-symmetric battery with **b** PFE-ALODS and **c** PFE-TPDS (The inserted figure is the impedance before and after the test, where the line is the equivalent circuit-fitting result). ^7^Li solid-state NMR of **d** PFE-ALODS before and after cycling and **e** PFE-TPDS before and after cycling. FT-IR spectra of the combination of two phases of each component. **g** Integration of the absorbance of the electrolyte in the carbonyl signal region. Changes in the content ratio of carbonyl coordination in **h** PFE-ALODS and **i** PFE-TPDS before and after cycling

Although surface acid–base interactions may not constitute the primary mechanism of lithium-ion transport, the surface effects of inorganic materials cannot be disregarded. Reducing particle size to enhance the specific surface area emerges as an effective strategy for improving material ionic conductivity. The experimental results reveal a fascinating progression of ionic conductivity with particle size modification. When alumina particle dimensions are reduced to the 200–300 nm range, the PFE-U-ALODS achieves an ionic conductivity of 0.12 mS cm^−1^ (Fig. 3a). Moreover, a further tenfold reduction in particle size enables the PFE-ALODS to reach an impressive ionic conductivity of 0.44 mS cm^−1^, a value proximate to that of LATP.

The specific surface area undoubtedly constitutes a critical factor influencing composite quasi-solid-state electrolytes. Assuming inorganic particles approximate spherical geometry, the specific surface area exhibits an inverse relationship with particle diameter. Consequently, smaller inorganic particles inevitably present more abundant surface interactions. A trend was observed wherein ionic conductivity consistently increases as aluminum oxide particle size is reduced from the micron scale (PFE-N-ALODS or PFE-A-ALODS) to the submicron scale (PFE-U-ALODS) and further to the nanoscale (PFE-ALODS). Concurrently, PFE-ALODS demonstrates a remarkable surge in lithium-ion transference number to 0.87 (Fig. 3b), indicating that lithium ions have become the predominant charge carriers in the bulk electrolyte’s ionic current conduction, while the mobility of lithium salt anions has been significantly restricted. Considering that lithium ions in liquid electrolytes are inherently surrounded by solvation shells that partially incorporate anions, these results suggest that anions have been constrained by certain interaction forces. Although it remains challenging to attribute this constraining effect entirely to the Lewis acid–base interactions previously discussed, these interactions undoubtedly originate from the inorganic particle surfaces, as evidenced by their strong correlation with particle size. The surface chemistry of the inorganic particles creates localized electronic environments that can effectively interact with and immobilize anions, thereby enhancing the relative contribution of lithium ions to the overall ionic conductivity of the composite electrolyte system.

### Active and Inert Fillers

There is a comparative analysis of two distinct inorganic materials between chemically inert alumina and the ionically conductive LATP. To ensure that the inorganic materials have similar reinforcing effects and to compare different mechanisms, we selected inorganic materials with different particle sizes. As shown in Fig. S5, the ionic conductivities of flexible membranes prepared with LATP and nanosized alumina (designated as PFE-TPDS and PFE-ALODS, respectively) exhibit different temperature-dependent behavior. The surface chemistry of both nano-alumina and LATP exhibits acidity due to surface-adsorbed hydroxyl groups [22, 37]. At room temperature, PFE-TPDS demonstrates a marginally superior ionic conductivity of 0.51 mS cm^−1^ compared to PFE-ALODS’s 0.44 mS cm^−1^ (Fig. 3a), substantiating the enhanced role of active inorganic materials in facilitating Li^+^ transportation. This superiority is further evidenced by the lower activation energy observed in the LATP-based system. When the temperature is higher, PFE-ALODS’s ionic conductivity approaches and occasionally surpasses that of PFE-TPDS. This unexpected behavior can be attributed to the nano-alumina’s smaller particle size, which results in a significantly larger specific surface area, where the enhanced surface area/volume ratio of the nano-alumina particles creates more extensive interfaces for ionic transportation. PFE-TPDS’s Li^+^ transference number is 0.50 (Fig. 3c). The observed inverse relationship between ionic conductivity and Li^+^ transference number points to a more complex surface interaction mechanism extending simple cation–anion anchoring effects.

The freshly prepared PFE-ALODS samples initially display a single ^7^Li NMR signal at -0.65 ppm in Fig. 3d, suggesting that the polymer matrix effectively incorporated all Li^+^ from the electrolyte, which is commonly observed in polymer-based quasi-solid-state electrolytes where the polymer-to-electrolyte ratio is relatively high, leading to extensive polymer–ion interactions [38]. There is a particular transformation after cycling that the original − 0.65 ppm peak undergoes splitting, resulting in two distinct signals at − 0.45 and − 0.95 ppm, revealing fundamental changes in the Li^+^ environments during cycling. For PFE-TPDS (Fig. 3e), before cycling, Li^+^ from the salt primarily exists in a state characterized by a − 0.62 ppm peak, reflecting a combined influence of liquid electrolyte and polymer interactions. After cycling, the low-field peaks become sharper, which suggests enhanced mobility of lithium ions within the liquid electrolyte environment. And the system exhibits a similar split pattern to PFE-ALODS, while alumina contains no lithium, LATP inherently peaks at − 0.96 ppm. Peak area integration reveals an increased relative lithium content at higher fields. This finding indicates that a significant portion of the lithium ions released from the polymer matrix have been adsorbed onto the surface of inorganic particles.

When examining binary component combinations (liquid electrolyte PFE–inorganic material LATP, inorganic material LATP–polymer DS, and liquid electrolyte PFE–polymer DS) as shown in Fig. 3f, carbonyl peaks are red-shifted from above 1700 cm^−1^ to about 1650 cm^−1^ in all systems containing LATP, compared to the single-component spectra displayed in Fig. S6. This spectral shift indicates significant interactions between the components that alter the carbonyl group’s chemical environment. The minimal spectral changes evidence the stability of both PFE-TPDS and PFE-ALODS systems before and after cycling (Fig. S7), except for the carbonyl peak region. The preservation of spectral features suggests that while the Li^+^ coordination structures undergo reorganization during electrochemical cycling, the overall structural integrity of the composite systems remains intact. The post-cycling spectrum shows a pronounced increase in carbonyl vibration peaks in the low-frequency region of the composite quasi-solid-state electrolyte in Fig. 3g, which correlates meaningfully with NMR, where Li^+^ signals exhibit an upfield shift. There are two concurrent phenomena: the Li^+^ chemical environment increasingly resembles that of the inorganic material, and the solvent carbonyl signals undergo a red shift due to confinement effects. This correlation suggests a mechanistic relationship: Li^+^ partially dissociates from their original environments (liquid electrolyte or polymer matrix) under electrochemical influence and migrates to the inorganic surface.

The semi-quantitative nature of FT-IR spectroscopy enables the attribution of peak integral area ratio changes (Fig. 3h, i). For alumina that lacks intrinsic Li^+^ conductivity, the composite effect manifests primarily through selective adsorption’s influence on electrolyte solvation structures, as evidenced by the dominance of solvents’ C=O. In contrast, LATP, being a lithium-rich phase with ionic conductivity, shows predominant carbonyl signals attributed to intermediate C=O, indicating the formation of new coordination structures at the composite interface under electrochemical conditions.

**Fig. 4:**
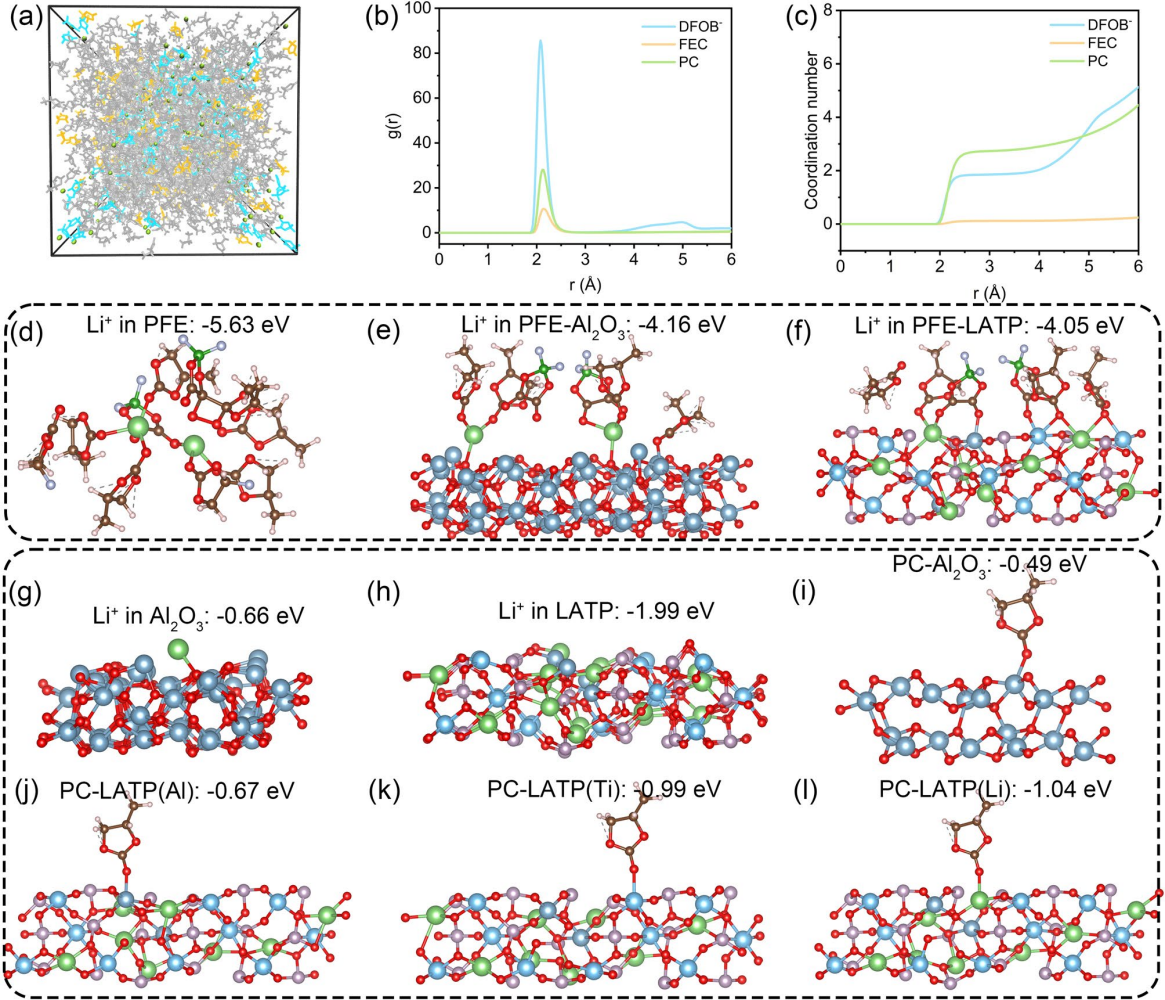
**a** Snapshot of the optimized solvated structure where Li^+^ is depicted in olive, PC in gray, DFOB^−^ in blue, and FEC in yellow. **b** Radial distribution function (g(r)) and **c** coordination number of solvation structure. **d-f** The binding energy of Li^+^ in solvent structure and surface structure. **g–h** The binding energy of Li^+^ on nano-alumina and LATP. **i-l** The binding energy of PC on different atoms of nano-alumina and LATP

To understand how the coordination environment influences ion transport, the molecular dynamics simulation provides crucial insights into the evolution of Li^+^ coordination structures across different environments. In the liquid electrolyte PFE, the primary solvation shell of Li^+^ exhibits a well-defined structure, as revealed by snapshots in Fig. 4a–c. Through careful analysis of radial distribution functions and coordination number calculations, we can see that the solvation shell is predominantly composed of two key components: PC (propylene carbonate), which is present in the highest concentration, and DFOB^−^ (difluoro(oxalato)borate), which demonstrates the strongest binding affinity. The strongest binding affinity of DFOB^−^ presents a significant challenge in LiDFOB’s complete dissociation within conventional electrolyte systems. Consequently, DFOB^−^ becomes intimately incorporated into the lithium ion’s solvation sheath, collaborating with PC (Fig. S8) to form CIPs (contact ion pairs) or AGGs (cation–anion aggregates) structural configurations.

This primary solvation shell creates a highly stable environment for the Li^+^ with a binding energy of − 5.63 eV (Fig. 4d). As Li^+^ partially dissociates and becomes adsorbed on the inert alumina surface, their binding energy decreases significantly to − 4.16 eV (Fig. 4e). When there is LATP, the binding energy further reduces to − 4.05 eV (Fig. 4f). From a thermodynamic perspective, lower binding energies typically indicate greater stability, which would suggest that Li^+^ should preferentially remain within their solvation shells under ambient environments. Lower binding energies facilitate more rapid ion transport when we consider electrochemical kinetics. This is why we see such dramatic changes in NMR and FT-IR signatures after cycling, with the system reorganizing to optimize dynamic transport rather than static stability. This creates a scenario in the intermediate layer, where Li^+^ experiences a hybrid solvation environment influenced by both the inorganic surface and the liquid electrolyte [39, 40]. When examining inorganic interfaces without considering solvent layers, the aluminum oxide surface lacks specific binding sites, presenting only negatively charged oxygen atoms for lithium-ion adsorption. LATP, with three-dimensional lithium-ion conduction channels, has a more complex surface structure. Beyond the thermodynamically stable M1 sites, the LATP crystal surface also features M2 sites, which correspond to lithium vacancies along the transport pathways [9]. These M2 sites have been demonstrated to exert significantly stronger attraction toward Li^+^ ions compared to M1 sites (Fig. 4g, h). Therefore, LATP’s strong surface interactions lead to a weakening of the traditional liquid electrolyte solvation effects.

To better understand how spatial confinement affects these interactions at the interface, PC was used as a model molecule for adsorption studies. The results shown in Fig. 4i–l reveal a hierarchy of interaction strengths that PC exhibits the weakest adsorption on alumina surfaces while showing significantly stronger interactions with LATP through two distinct mechanisms of anchoring to Ti sites and coordination with Li atoms. All these complex interactions at the interface produce several important effects. First, the negative charge normally concentrated in liquid electrolyte molecules becomes more dispersed when they interact with the interface, weakening coordination effects within the intermediate layer. Second, LATP’s stronger affinity for PC molecules helps facilitate the migration of coordinated solvation structures and promotes partial dissociation of Li^+^. These results help explain why PFE-TPDS shows superior ion transport properties compared to PFE-ALODS, with stronger surface interactions helping to create more favorable pathways for ion transport by modifying the local coordination environment in beneficial ways. During the X-ray photoelectron spectroscopy (XPS, Fig. S9) surface compositional analysis of the pristine PFE-TPDS, we serendipitously detected an unexpected signal of approximately 15% low-valence titanium. This observation aligns with previous works documenting the potential surface adsorption of liquid electrolytes and their degradation products on oxide surfaces [41]. When the system comprised solely propylene carbonate (PC) and LATP, the surface still exhibited approximately 12% low-valence titanium, which directly corresponds to the influence of residual solvent adsorption. This phenomenon becomes more pronounced in the bulk phase where PC demonstrates reduced volatility. However, considering the relatively higher proportion of low-valence titanium on the PFE-TPDS surface compared to the PC-LATP system, we postulate that a portion of this signal can be attributed to lithium-ion surface interactions and binding.

### Interfacial Electrochemical Stability

**Fig. 5:**
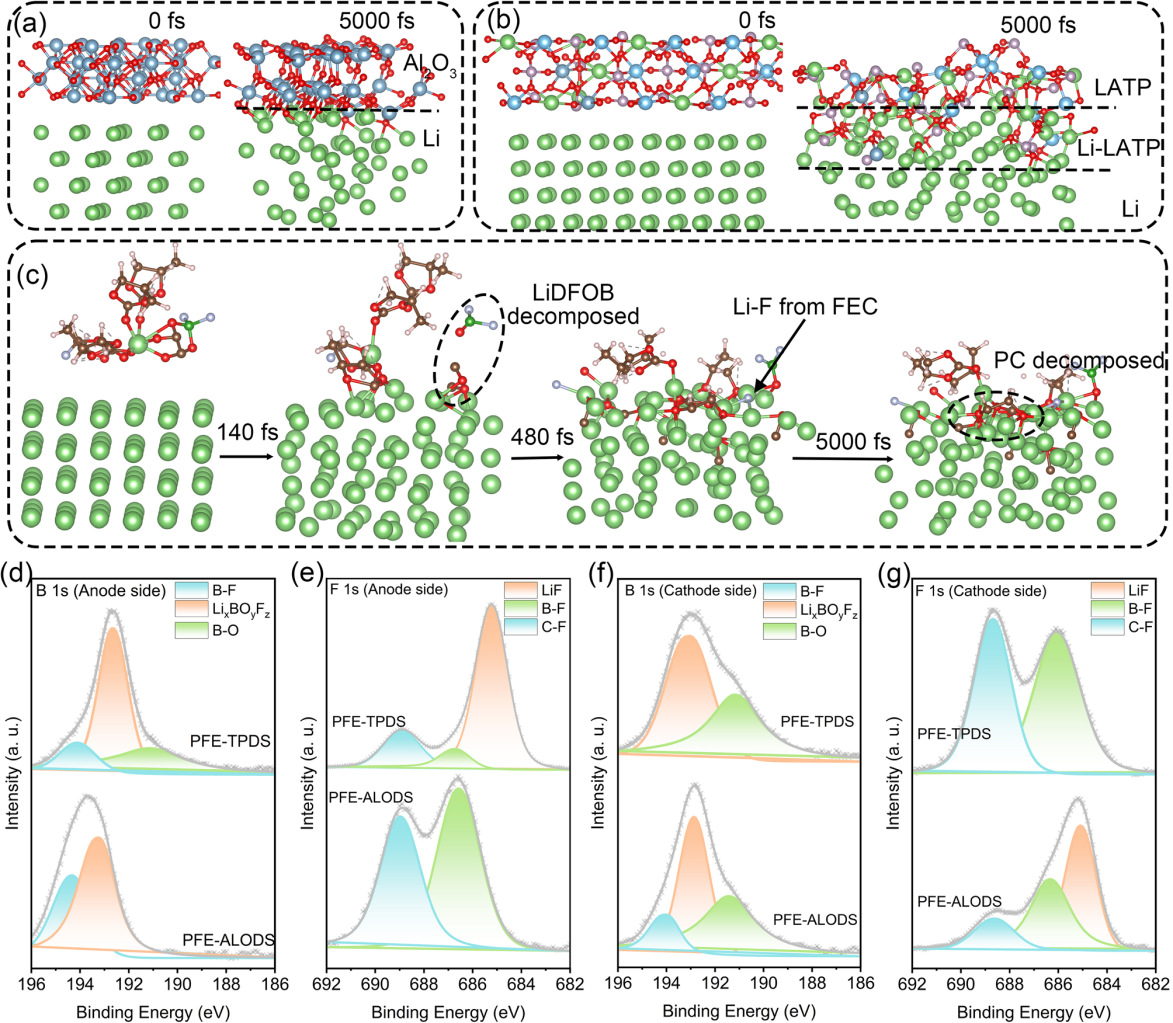
Snapshots of AIMD simulation results in **a** lithium metal/nano-alumina, **b** lithium metal/LATP, and **c** lithium metal/PFE solvent. **d** B 1*s* and **e** F 1*s* spectra of electrolyte in the anode side. **f** B 1*s* and **g** F 1*s* spectra of electrolyte in the cathode side

The ab initio molecular dynamics (AIMD) simulation results at the lithium metal interface demonstrated that aluminum oxide maintains its structural integrity at the interface, which is indicative of its superior stability against the lithium anode (Fig. 5a). When LATP comes into direct contact with the lithium anode, being a strong reducing agent, a continuous reduction of Ti^4+^ ions is initiated. It was evidenced in AIMD simulations, which reveal the formation of an undesirable mixed reaction interface layer (Fig. 5b). Fortunately, in the case of PFE, the formation of a stable solid electrolyte interphase (SEI) at the anode interface is achieved through the sequential decomposition of DFOB-, FEC (fluoroethylene carbonate), and PC (Fig. 5c).

Analysis of the anode interface components provided valuable insights into how different inorganic materials influence SEI. Alumina, characterized by its chemical inertness and electronic insulating properties, exhibits exceptional interfacial stability with lithium metal anodes. This indicates that aluminum oxide itself can function as a stable constituent of SEI, consequently reducing the decomposition of other electrolyte components. This phenomenon is evidenced by the substantial retention of B-F bonds observed in Fig. 5d, e. The absence of corresponding signals in the Ti 2*p* spectra following PFE-TPDS cycling (Fig. S10) suggests the formation of a thicker alternative SEI layer on the PFE-TPDS surface. This observation aligns with the understanding that reduction reactions between LATP and lithium metal proceed continuously and irreversibly. When LATP initially in direct contact with lithium metal undergoes reduction, the liquid electrolyte and lithium salt proximal to the anode become deanchored and subsequently participate in SEI formation. This process ultimately culminates in passivation, wherein an interfacial deposit primarily composed of LiF and Li_x_BO_y_F_z_ forms on the electrolyte membrane surface, thereby preventing further degradation of LATP. The liquid electrolyte in PFE-ALODS demonstrates less participation in interfacial reactions, maintaining an electrochemical window of 4.98 V (Fig. S11). Notably, PFE-TPDS achieves an electrochemical window of 5.08 V, comparable to that of the pristine liquid electrolyte (5.07 V), owing to the formation of a more stable SEI. This voltage range is theoretically sufficient to accommodate the operational voltage window of LiNi_0.5_Mn_1.5_O_4_ (LNMO) cathode. Analysis of the B 1* s* spectra (Fig. 5f) reveals that while PFE-TPDS lacks the B–F bonds characteristic of LiDFOB, it exhibits the presence of primary decomposition products (B–O bonds of LiBOB and Li_*x*_BO_*y*_F_*z*_) that maintain stability while facilitating charge transport. The cathode interface of PFE-ALODS, when examined in conjunction with F 1* s* spectra (Fig. 5g), indicates the presence of the ultimate decomposition product, electrically insulating LiF.

**Fig. 6:**
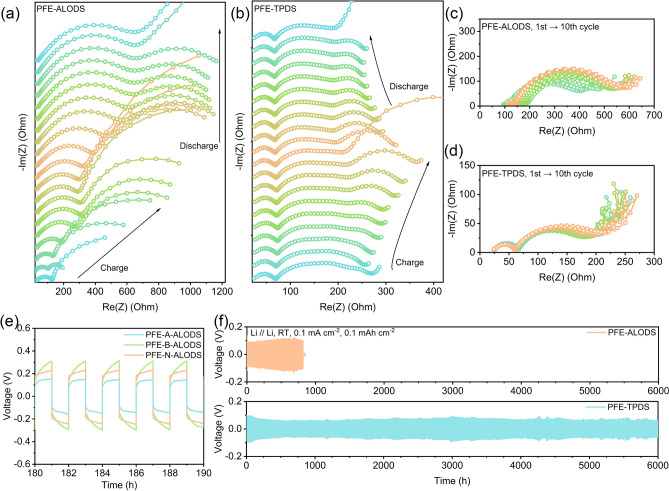
In situ electrochemical impedance spectra of a single cycle in **a** LNMO / PFE-ALODS / Li and **b** LNMO / PFE-TPDS / Li. **c-d** Impedance changes of these batteries from the first to the tenth cycle. **e** Partially enlarged lithium-symmetric battery cycle curves during 180–190 h for nano-alumina with different surface acidity and alkalinity. **f** Lithium-symmetric battery cycle curves of PFE-ALODS and PFE-TPDS

The in situ EIS measurements during the initial cycle of LNMO//Li cells utilizing PFE-ALODS and PFE-TPDS electrolytes are presented in Fig. 6a, b. During the charging process, both systems exhibit a similar impedance evolution pattern, wherein initial interface wetting and activation lead to impedance reduction, followed by an increase attributed to interfacial decomposition reactions. The inferior cathode stability of PFE-ALODS manifests in a double impedance growth as the voltage increases, whereas PFE-TPDS demonstrates a more gradual impedance evolution, with notably stable high-frequency impedance components. During the discharge process, PFE-TPDS exhibits excellent reversibility, with impedance values progressively returning to levels comparable to those observed before cycling. Although PFE-ALODS maintains a certain impedance magnitude during voltage reduction, it fails to demonstrate a decreasing trend.

Comparative analysis of impedance evolution over the first ten charge–discharge cycles (Fig. 6c, d) reveals rapid impedance growth in PFE-ALODS. Distribution of relaxation times (DRT) analysis was employed to establish the correlation between impedance and time constants. As evidenced in Fig. S12, the predominant impedance increase occurs within the relaxation time (*τ*) range of 10^–4^ to 10^–2^ s, which precisely corresponds to the cathode electrolyte interphase (CEI) layer. The negligible impedance variations observed in PFE-TPDS substantiate its superior stability in 5 V-class cathode full cells.

### Electrochemical Performance

Given that the acid–base characteristics of the aluminum oxide interface operate independently of ionic conductivity, the progressive voltage increase observed within individual cycles of lithium-symmetric cells can be attributed to concentration polarization phenomena. The enhancement of interfacial basicity results in more challenging dissociation of solvation structures, consequently leading to heightened concentration polarization effects. Under conditions of comparable ionic conductivity, acidic interfaces facilitate Li^+^ dissociation more effectively, thereby enabling the maintenance of stable polarization within lithium-symmetric cells (Figs. S13 and 6e). As the specific surface area of inorganic materials increases, a corresponding enhancement in ionic conductivity is observed, which subsequently results in the reduction of polarization voltage to levels below 100 mV (Figs. 6f and S14). The distinct behavioral patterns of the two systems are particularly evident in their long-term stability characteristics. Though PFE-ALODS, exhibiting reduced degradation at the anode, demonstrates increased vulnerability to lithium dendrite propagation along the inorganic material interfaces, ultimately leading to short-circuit failure after 817 h. In contrast, PFE-TPDS maintains operational integrity without short-circuiting for an extended duration of 6000 h, providing compelling evidence for the enhanced stability of its SEI.

**Fig. 7:**
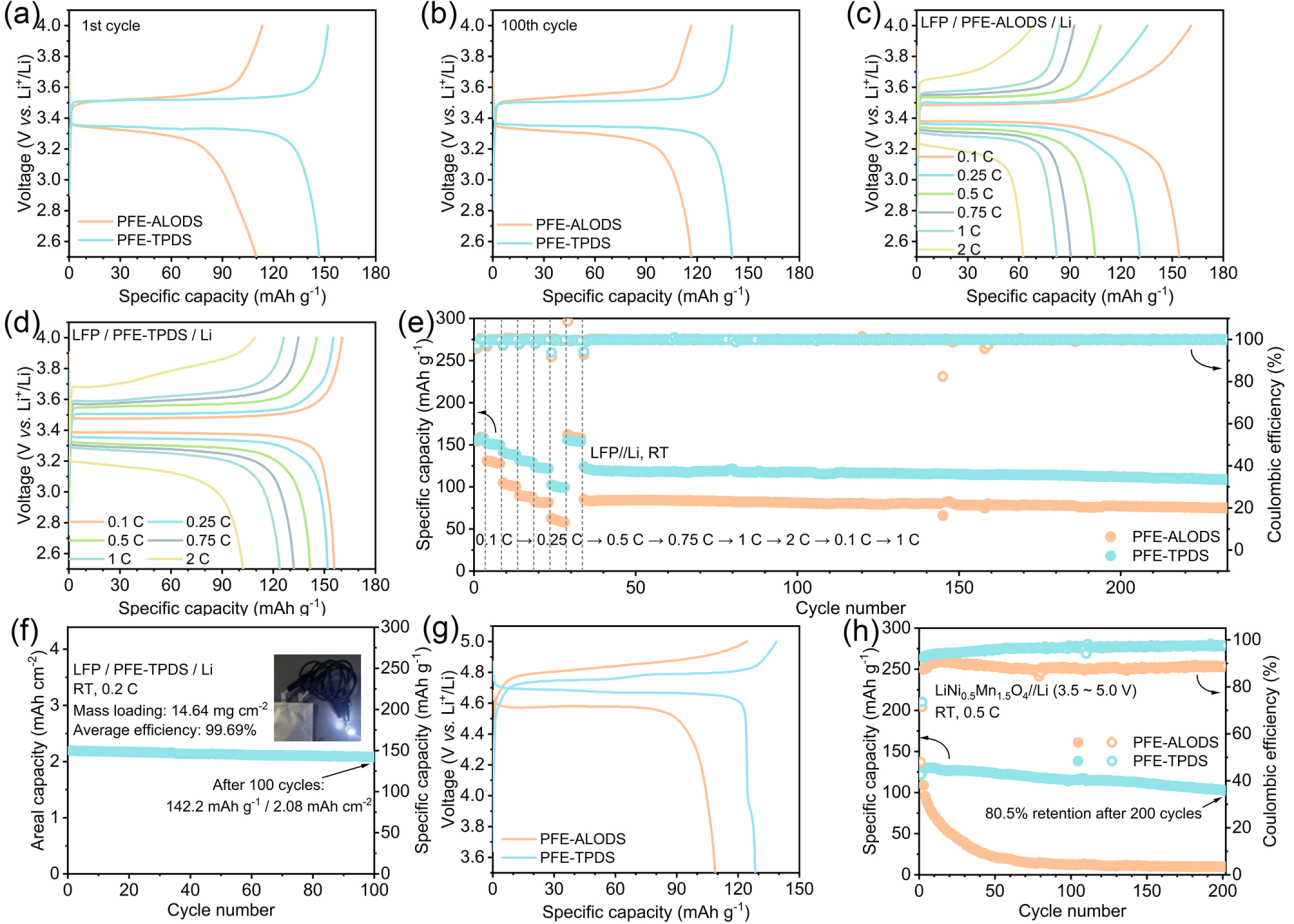
The charge and discharge curves of **a** the first and **b** the 100th cycle in LFP//Li cells. The charge and discharge curves of **c** PFE-ALODS and **d** PFE-TPDS in LFP//Li cells at different rates. **e** Cycling performance of switching different rates of PFE-ALODS and PFE-TPDS in LFP//Li cells. **f** Cycling performance in a high-loading mass of PFE-TPDS where the inserted picture shows a photograph of a small pouch battery lighting up an LED. **g** The first charge and discharge curves and **h** cycling performance of PFE-ALODS and PFE-TPDS in LNMO//Li cells

The enhanced ionic conductivity of quasi-solid-state electrolytes manifests in superior specific capacity performance within lithium metal batteries. When employing the chemically stable LFP (LiFePO_4_) as the cathode material, both PFE-TPDS and PFE-ALODS demonstrate stable cycling performance at 0.5C (Fig. S15a, b). However, after 100 cycles, cells incorporating PFE-TPDS as the separator maintain a discharge-specific capacity of 140 mAh g^−1^, which substantially exceeds the 110 mAh g^−1^ observed with PFE-ALODS (Fig. 7a, b). This performance distinction becomes more pronounced across various rates. PFE-ALODS delivers discharge-specific capacities of 154.2, 131.0, 105.0, 90.5, 82.2, and 62.6 mAh g^−1^ at 0.1C, 0.25C, 0.5C, 0.75C, 1C, and 2C, respectively (Fig. 7c). In contrast, PFE-TPDS exhibits superior performance with discharge-specific capacities of 155.8, 151.9, 141.7, 132.2, 123.9, and 102.4 mAh g^−1^ under identical testing conditions (Fig. 7d). The excellent anodic electrochemical stability of both quasi-solid-state electrolytes enables reversible capacity retention at high rates, maintaining stable capacity throughout rate capability tests (Fig. 7e). Notably, when the cathode loading is increased to 14 mg cm^−2^, a remarkable discharge-specific capacity of 142.2 mAh g^−1^ is retained after 100 cycles (Fig. 7f). The practical applicability of these systems was demonstrated through the successful fabrication of pouch cells capable of powering light-emitting diode (LED) displays and driving higher-power motor fans (Video S1).

When transitioning to LiNi_0.6_Co_0.2_Mn_0.2_O_2_ cathodes with a cutoff voltage of 4.3 V (Fig. S16a, b), capacity performance patterns like LFP are observed, with the higher ionic conductivity PFE-TPDS delivering a discharge-specific capacity exceeding that of PFE-ALODS by 23 mAh g^−1^. Despite increased cathode interface instability introducing cycling uncertainties in full cells, PFE-TPDS demonstrates superior performance with high-voltage cathodes, consistent with previous characterization results. After 100 cycles at 0.5C, it retains 90.2% of its initial discharge-specific capacity. This performance advantage becomes even more pronounced with 5 V-class LNMO cathodes (Fig. 7g, h), where PFE-TPDS achieves an initial discharge-specific capacity of 128.4 mAh g^−1^ and maintains an impressive capacity retention of 80.5% after 200 cycles at 0.5C, significantly outperforming PFE-ALODS. These results conclusively demonstrate the exceptional performance capabilities of quasi-solid-state electrolytes fabricated with high LATP content.

## Conclusions

In this work, the acid–base properties of inorganic surfaces do not substantially influence ionic conductivity; instead, their primary contribution lies in facilitating selective interfacial adsorption, which promotes the dissociation of solvation structures. The observation that ionic conductivity can be effectively enhanced through increased specific surface area and modifications to inorganic fillers points to the critical role of interfacial phenomena within composite quasi-solid-state electrolytes. When comparing active and inert oxides, both demonstrate the ability to reorganize Li^+^ at their surfaces into coordinated interfacial structures. This reorganization promotes partial Li^+^ dissociation and rapid conduction. However, LATP, possessing inherent ionic conductivity, offers kinetically favorable interfacial coordination, ultimately achieving a superior ionic conductivity of 0.51 mS cm^−1^. The inorganic components create a network of interfaces that facilitate ion transport and contribute to forming stable electrode interfaces at high-voltage cathodes and lithium metal anodes. LFP//Li batteries maintain a high discharge-specific capacity of 142.2 mAh g^−1^ even under elevated cathode loading conditions. More impressively, when implemented in LNMO//Li batteries operating at 0.5C, the system demonstrates exceptional stability with a capacity retention of 80.5% after 200 cycles. These findings provide guidance for the development of inorganics at the conducting interface and electrode interface in composite quasi-solid-state battery technology.

## Supplementary Information

Below is the link to the electronic supplementary material.

Supplementary file1 (DOCX 1754 KB)

Supplementary file2 (MP4 13765 KB)
