# Supplementary material for: Enhancement of Li+ Transport Through Intermediate Phase in High-Content Inorganic Composite Quasi-Solid-State Electrolytes
Source: Nanomicro Lett. 2025 Jun 11;17:288. doi: 10.1007/s40820-025-01774-5 (PMC12158885; doi:10.1007/s40820-025-01774-5)
Supplement: Supplementary file 1 — Supplementary file1 (DOCX 1754 KB) [file 40820_2025_1774_MOESM1_ESM.docx]

Supporting Information for

**Enhancement of Li^+^ Transport through Intermediate Phase in High-Content Inorganic Composite Quasi-Solid-State Electrolytes**

Haoyang Yuan^1^, Wenjun Lin^2^, Changhao Tian^1^, Mihaela Buga^3,^ *, Tao Huang^2,^ *, and Aishui Yu^1, 2,^ *

^1^ Department of Chemistry, Collaborative Innovation Center of Chemistry for Energy Materials, Shanghai Key Laboratory of Molecular Catalysis and Innovative Materials, Institute of New Energy, Fudan University, Shanghai 200438, P. R. China

^2^ Laboratory of Advanced Materials, Fudan University, Shanghai 200438, P. R. China

^3^ ROM-EST Laboratory, ICSI Energy Department, National Research and Development Institute for Cryogenic and Isotopic Technologies − ICSI, 4 Uzinei, Ramnicu Valcea 240050, Romania

*Corresponding authors. E-mail: [mihaela.buga@icsi.ro](mailto:mihaela.buga@icsi.ro) (Mihaela Buga); [huangt@fudan.edu.cn](mailto:huangt@fudan.edu.cn) (Tao Huang); [asyu@fudan.edu.cn](mailto:asyu@fudan.edu.cn) (Aishui Yu)

**S1 Experimental section**

**S1.1 Materials characterization**

The structural and chemical characterization of the electrolytes was conducted using multiple complementary analytical techniques: X-ray diffraction (XRD, Bruker D8 Advance) was employed to analyze the crystalline phases of the inorganic components. Organic functional groups were qualitatively identified using Fourier transform infrared spectroscopy (FT-IR, Thermo Fisher Scientific Nicolet iS50) equipped with attenuated total reflection (ATR). The lithium-ion coordination environment was investigated through magic angle spinning (MAS) solid-state nuclear magnetic resonance (NMR, 400WB AVANCE III) spectroscopy. Scanning electron microscopy (SEM, Hitachi-S4800) coupled with energy-dispersive X-ray spectroscopy (EDS, Oxford X-MAX 80T) was utilized to examine the morphology and thickness of the inorganic materials and electrolyte layers. Interface compositional analysis was performed using X-ray photoelectron spectroscopy (XPS, Thermo Scientific ESCALAB Xi+) integrated with a glovebox vacuum transfer system to prevent sample contamination.

**S1.2 Computational methods**

All quantum mechanical calculations were performed using a combination of different software packages. The initial structure optimization of the organic molecules was carried out using Orca 5.0.4, where the B3LYP-D3(BJ) functional with the def2-TZVP basis set was employed to optimize the molecular geometry [S1-S9]. To analyze the solvation effects, the solute molecules were treated with explicit solvent models using GROMACS 2022.5 [S10]. Two different force fields, the Generalized Amber Force Field (GAFF) and the Universal Force Field (UFF), were employed to represent the solute and solvent interactions [S11, S12]. The force field parameters for both solute and solvent were obtained from Sobtop [S13]. The initial system was energy minimized using the steepest descent algorithm to remove any steric clashes and relax the system to a stable configuration. The system was equilibrated in the canonical ensemble (NVT) at 300 K using a Nose-Hoover thermostat [S14]. A 100 ps equilibration time was used to stabilize the system's temperature and volume. After NVT equilibration, the system was further equilibrated in the isothermal-isobaric ensemble (NPT) to allow for density relaxation and volume stabilization. The pressure was maintained at 1 bar using the Parrinello-Rahman barostat over a 5 ns period [S15]. After the equilibration steps, a 5 ns production MD simulation was carried out in the NPT ensemble, with the system's temperature controlled at 300 K and pressure at 1 bar. A time step of 1 fs was used for simulation, and data was collected for analysis of radial distribution functions (RDF, g(r)) and coordination number (CN) by these equations.

$g\left( r \right)={{dn\left( r \right)}/{dr}}/{4\pi r^{2}\rho}$ (S1)

$CN=4\pi\rho\int_{0}^{r} r^{2}g\left( r \right) dr$ (S2)

where r is the distance of particles and Li^+^ in the system, ρ is average particle density, and dn(r) is the total number of particles within the thickness dr.

CP2K 2023.1 was used for further geometry optimization and binding energy calculations [S16]. The PBE functional was employed within the DFT framework, using the DZVP-MOLOPT-SR-GTH basis set for valence electrons [S17, S18]. The system was optimized under periodic boundary conditions, with the XY being periodic, and the energy cutoffs were set to 400 Ry for the wavefunction, ensuring accurate treatment of the electronic structure. Binding energies were calculated by taking the energy difference between the complex and the isolated components. For dynamic simulations, ab initio molecular dynamics (AIMD) was carried out using the Born-Oppenheimer approximation with a time step of 1.0 fs. The system temperature was maintained at 300 K using a Nose-Hoover thermostat, and the resulting trajectories were analyzed to explore the system's structural evolution and energy behavior [S14]. Multiwfn and VMD were used for visualization [S19-S21].

**S2 Supplementary Figures**


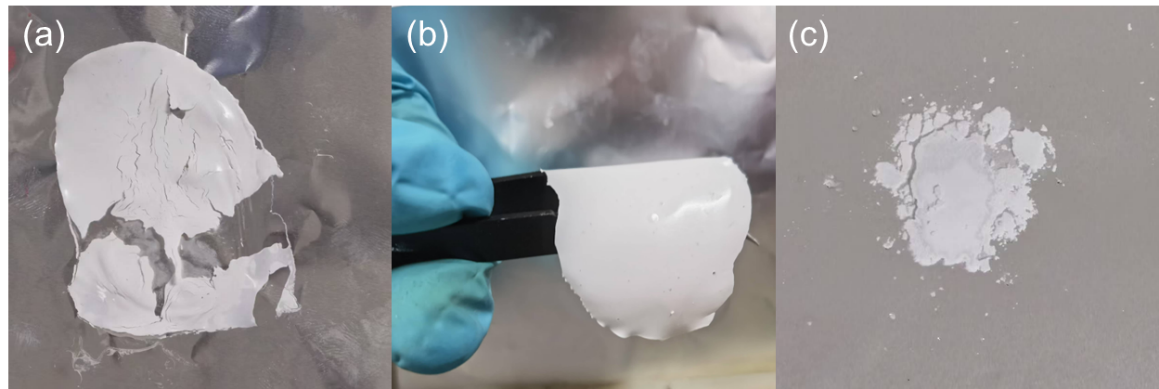


**Fig. S1** Optical photos of electrolyte membranes prepared at different ratios (LATP:liquid electrolytes:polymer): (**a**) 5:4:1, (**b**) 6:3:1, and (**c**) 7:2:1


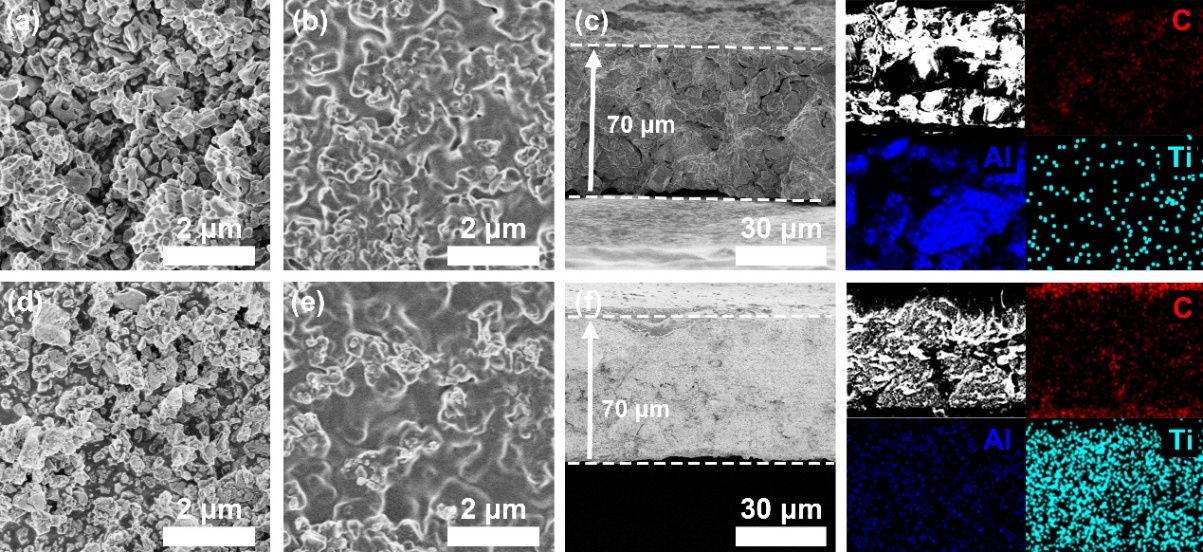


**Fig. S2** SEM images of (**a**) nano-alumina, (**b**) front, (**c**) cross-section and EDS mapping of PFE-ALODS, (**d**) LATP, (**e**) front, (**f**) cross-section and EDS mapping of PFE-TPDS


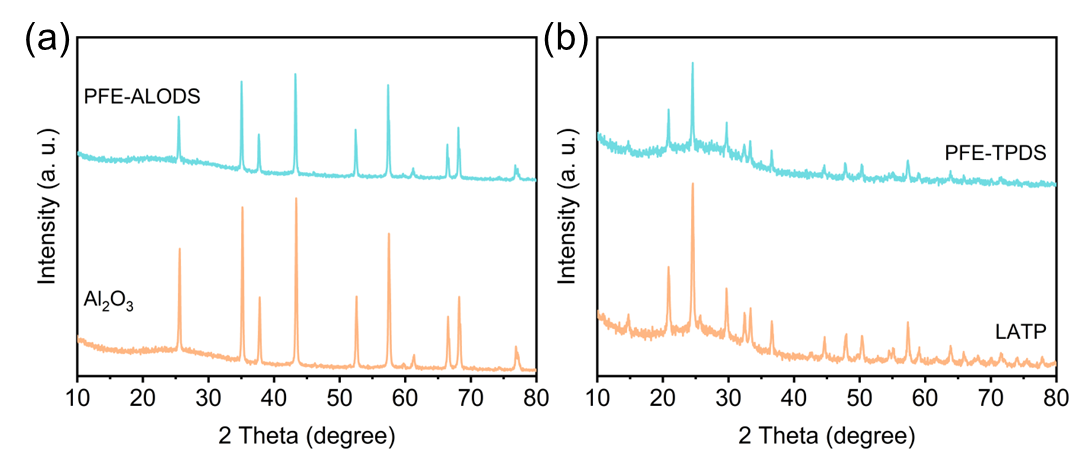


**Fig. S3** XRD spectra of (**a**) PFE-ALODS and (**b**) PFE-TPDS


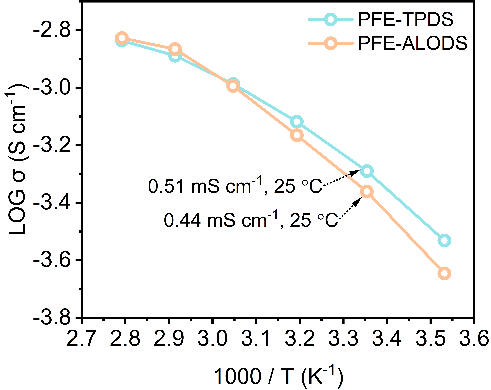


**Fig. S4** Variation trend of ionic conductivity of PFE-ALODS and PFE-TPDS with temperature


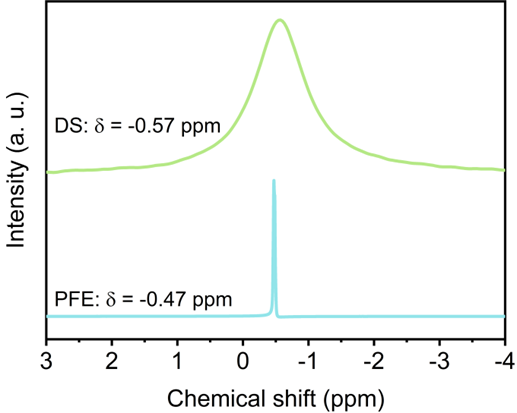


**Fig. S5** Solid state NMR spectra of liquid electrolyte PFE and polymer DS


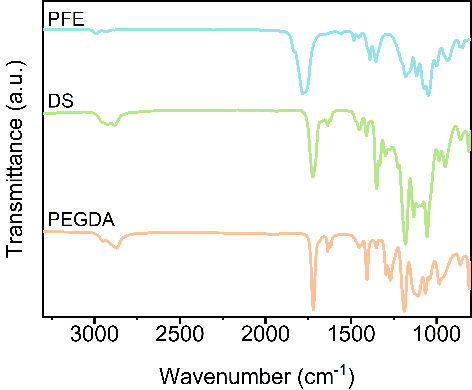


**Fig. S6** FT-IR spectra of every single material of the electrolyte


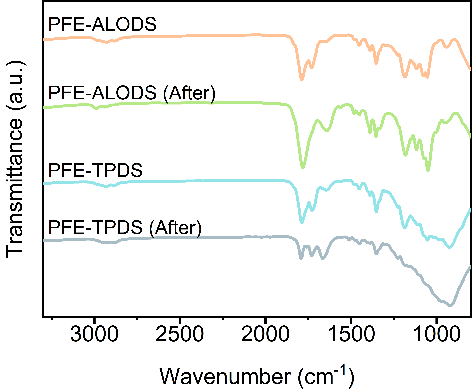


**Fig. S7** FT-IR spectra of PFE-ALODS and PFE-TPDS before and after cycling


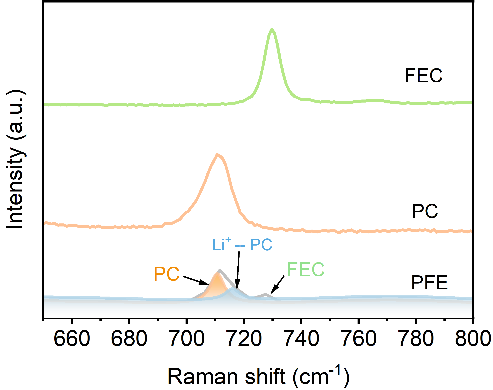


**Fig. S8** Raman spectra of FEC, PC, and PFE


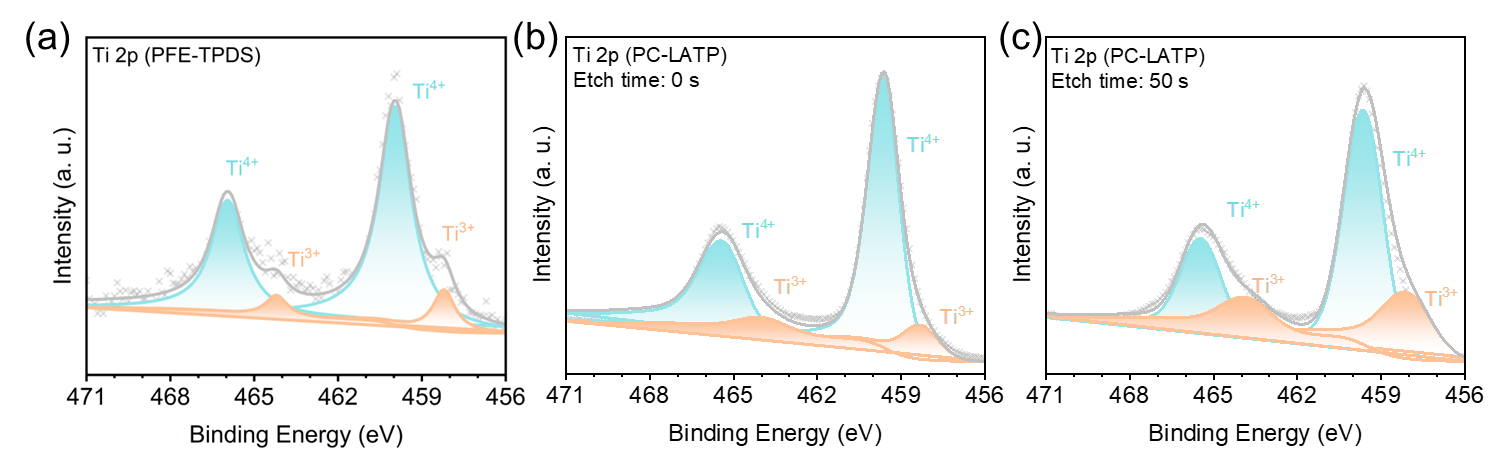


**Fig. S9** Ti 2p spectra of (**a**) PFE-TPDS, (**b**) PC-LATP surface, and (**c**) PC-LATP bulk

**Fig. S10** Ti 2p spectrum of PFE-TPDS after cycling on the anode side


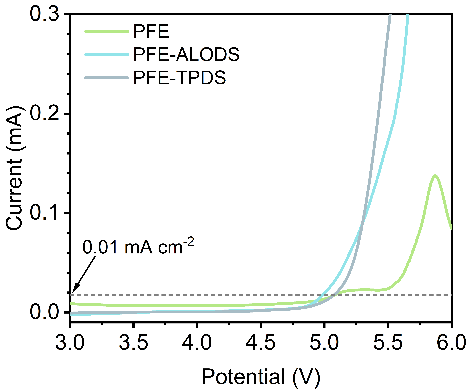


**Fig. S11** LSV curves of liquid electrolyte, PFE-ALODS and PFE-TPDS


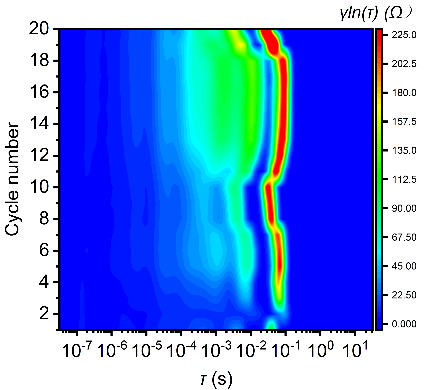


**Fig. S12** Three-dimensional contour diagram projection of DRT after the 1st to 10th electrochemical cycles of PFE-ALODS


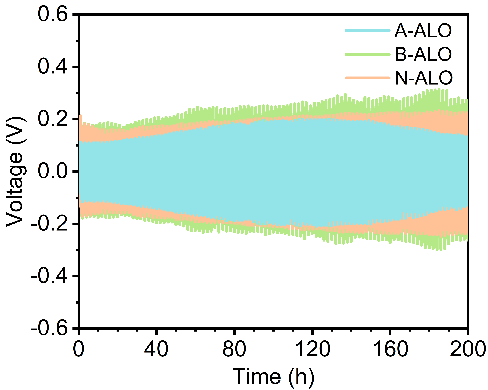


**Fig. S13** Voltage curves of quasi-solid electrolytes of different acidic and basic alumina in lithium symmetrical batteries in 0.1 mA cm^-2^


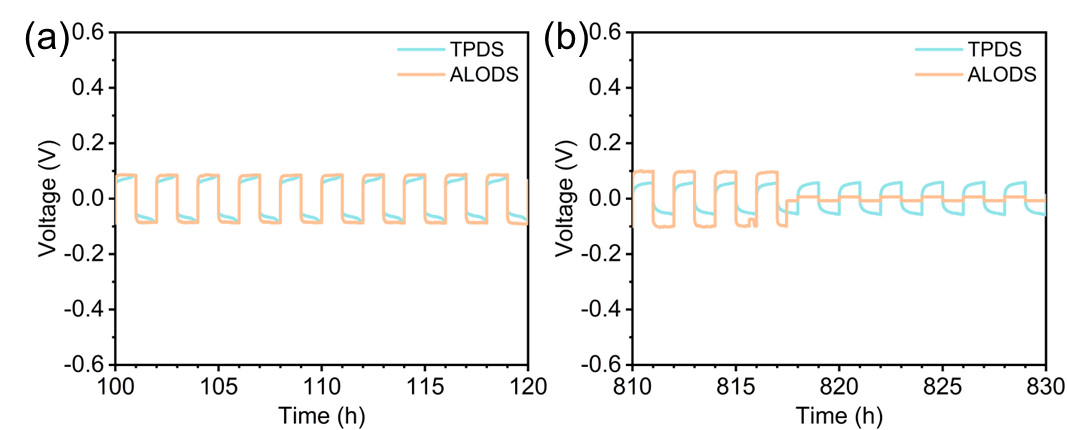


**Fig. S14** Lithium symmetric battery cycle curves of PFE-ALODS and PFE-TPDS in (**a**) 100-120 h and (**b**) 810-830 h


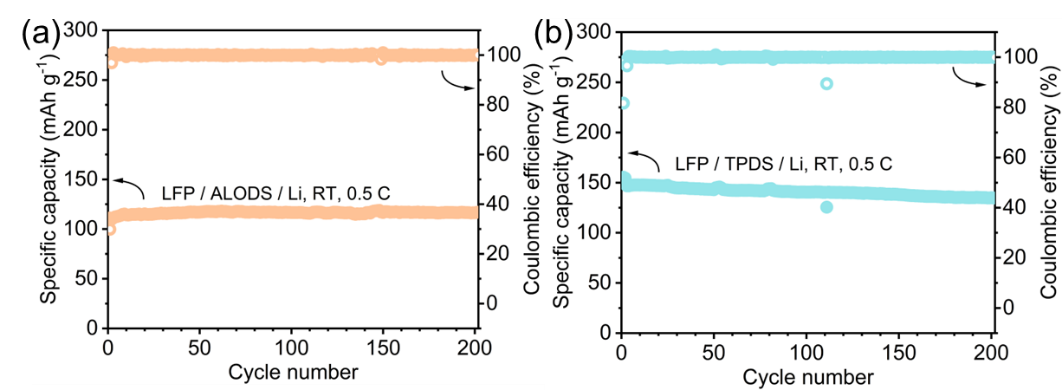


**Fig. S15** LFP // Li cells cycling performance using (**a**) PFE-ALODS and (**b**) PFE-TPDS


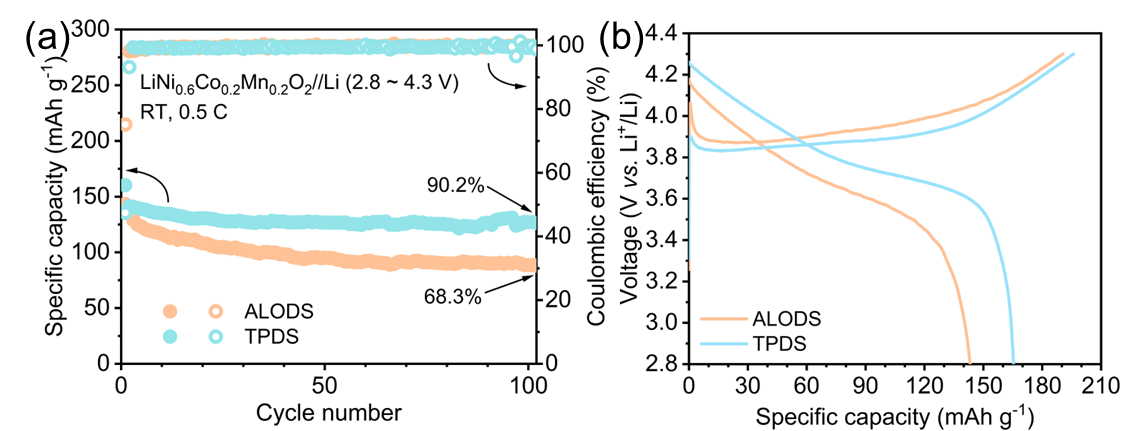


**Fig. S16** (**a**) Cycling performance and (**b**) charge-discharge curves of two quasi-solid electrolytes in the first cycle with NCM622 as cathode

Supplementary References

1. F. Neese, F. Wennmohs, U. Becker, C. Riplinger, The *ORCA* quantum chemistry program package. J. Chem. Phys. **152**(22), 224108 (2020). <https://doi.org/10.1063/5.0004608>
2. F. Neese, Software update: the *ORCA* program system: version 5.0. Wires Comput. Mol. Sci. **12**(5), e1606 (2022). <https://doi.org/10.1002/wcms.1606>
3. S. Grimme, J. Antony, S. Ehrlich, H. Krieg, A consistent and accurate *ab initio* parametrization of density functional dispersion correction (DFT-D) for the 94 elements H-Pu. J. Chem. Phys. **132**(15), 154104 (2010). <https://doi.org/10.1063/1.3382344>
4. S. Grimme, S. Ehrlich, L. Goerigk, Effect of the damping function in dispersion corrected density functional theory. J. Comput. Chem. **32**(7), 1456–1465 (2011). <https://doi.org/10.1002/jcc.21759>
5. R. Izsák, F. Neese, An overlap fitted chain of spheres exchange method. J. Chem. Phys. **135**(14), 144105 (2011). <https://doi.org/10.1063/1.3646921>
6. A.D. Becke, Density‐functional thermochemistry. III. The role of exact exchange. J. Chem. Phys. **98**(7), 5648–5652 (1993). <https://doi.org/10.1063/1.464913>
7. C. Lee, W. Yang, R.G. Parr, Development of the *Colle*-Salvetti correlation-energy formula into a functional of the electron density. Phys. Rev. B Condens. Matter **37**(2), 785–789 (1988). <https://doi.org/10.1103/physrevb.37.785>
8. F. Weigend, R. Ahlrichs, Balanced basis sets of split valence, triple *Zeta* valence and quadruple *Zeta* valence quality for H to Rn: Design and assessment of accuracy. Phys. Chem. Chem. Phys. **7**(18), 3297–3305 (2005). <https://doi.org/10.1039/B508541A>
9. F. Weigend, Accurate coulomb-fitting basis sets for H to Rn. Phys. Chem. Chem. Phys. **8**(9), 1057–1065 (2006). <https://doi.org/10.1039/B515623H>
10. H.J.C. Berendsen, D. van der Spoel, R. van Drunen, GROMACS: a message-passing parallel molecular dynamics implementation. Comput. Phys. Commun. **91**(1–3), 43–56 (1995). <https://doi.org/10.1016/0010-4655(95)00042-E>
11. J. Wang, R.M. Wolf, J.W. Caldwell, P.A. Kollman, D.A. Case, Development and testing of a general amber force field. J. Comput. Chem. **25**(9), 1157–1174 (2004). <https://doi.org/10.1002/jcc.20035>
12. A.K. Rappe, C.J. Casewit, K.S. Colwell, W.A. Goddard III, W.M. Skiff, UFF, a full periodic table force field for molecular mechanics and molecular dynamics simulations. J. Am. Chem. Soc. **114**(25), 10024–10035 (1992). <https://doi.org/10.1021/ja00051a040>
13. T. Lu, Sobtop, Version 1.0 (dev3.1), <http://sobereva.com/soft/Sobtop> (accessed: July 2023).
14. H.A. Posch, W.G. Hoover, F.J. Vesely, Canonical dynamics of the nosé oscillator: stability, order, and chaos. Phys. Rev. A Gen. Phys. **33**(6), 4253–4265 (1986). <https://doi.org/10.1103/physreva.33.4253>
15. M. Parrinello, A. Rahman, Polymorphic transitions in single crystals: a new molecular dynamics method. J. Appl. Phys. **52**(12), 7182–7190 (1981). <https://doi.org/10.1063/1.328693>
16. T.D. Kühne, M. Iannuzzi, M. Del Ben, V.V. Rybkin, P. Seewald et al., CP2K: an electronic structure and molecular dynamics software package - quickstep: efficient and accurate electronic structure calculations. J. Chem. Phys. **152**(19), 194103 (2020). <https://doi.org/10.1063/5.0007045>
17. S. Goedecker, M. Teter, J. Hutter, Separable dual-space Gaussian pseudopotentials. Phys. Rev. B Condens. Matter **54**(3), 1703–1710 (1996). <https://doi.org/10.1103/physrevb.54.1703>
18. C. Hartwigsen, S. Goedecker, J. Hutter, Relativistic separable dual-space Gaussian pseudopotentials from H to Rn. Phys. Rev. B **58**(7), 3641–3662 (1998). <https://doi.org/10.1103/physrevb.58.3641>
19. K. Momma, F. Izumi, VESTA 3 for three-dimensional visualization of crystal, volumetric and morphology data. J. Appl. Crystallogr. **44**(6), 1272–1276 (2011). <https://doi.org/10.1107/S0021889811038970>
20. W. Humphrey, A. Dalke, K. Schulten, VMD: Visual molecular dynamics. J. Mol. Graph. **14**(1), 33–38 (1996). <https://doi.org/10.1016/0263-7855(96)00018-5>
21. T. Lu, F. Chen, Multiwfn: a multifunctional wavefunction analyzer. J. Comput. Chem. **33**(5), 580–592 (2012). <https://doi.org/10.1002/jcc.22885>
